# What influences physician opioid prescribing for children with acute pain?

**DOI:** 10.1177/20494637221146421

**Published:** 2022-12-15

**Authors:** George Slim, Michael van Manen, Megan Fowler, Naveen Poonai, Samina Ali

**Affiliations:** 1 Department of Pediatrics, Faculty of Medicine & Dentistry, 3158 University of Alberta, Edmonton, AB, Canada; 2 Women and Children Health Research Institute, 3158 University of Alberta, Edmonton, AB, Canada; 3 Department of Emergency Medicine, Section of Pediatric Emergency Medicine, Schulich School of Medicine and Dentistry, 6221 Western University, London, ON, Canada

**Keywords:** qualitative, healthcare professionals, narcotics, opinions

## Abstract

**Background:**

Pain is one of the most common symptoms encountered in the healthcare system, and opioids are among the top three medications used to treat it. Understanding the reasoning behind physicians’ opioid prescribing practices is vital to safe practice. The primary objective of our study was to describe pediatric emergency physicians’ decision-making process when prescribing opioids for children’s acute pain management.

**Methods:**

This study employed qualitative methodology, using one-on-one semi-structured interviews within a grounded theory analytic framework. We employed purposeful sampling to recruit pediatric emergency physicians from across Canada. Interviews were conducted by telephone (December 2019–January 2021). Transcript analysis occurred concurrently with data collection, supporting data saturation and theory development considerations.

**Results:**

Eleven interviews were completed with participants representing each of Canada’s geographic regions. Nine major themes emerged: (1) practice setting and outpatient opioid use, (2) condition-specific considerations, (3) physician confidence in medical evidence, (4) pain assessment challenges, (5) patient and family perspectives, (6) opioid safety concerns, (7) personal biases and experiences, (8) personal practice context, and (9) the Opioid Crisis/media influence. Most clinicians felt that they limited opioid use to those who needed it most; all participants described challenges managing acute pain, emphasizing the need for accurate pain measurement and better guidelines, evidence-based data, and knowledge translation. Clinicians were more comfortable treating pain in the emergency department, compared to discharge prescribing. They recognized the importance of co-therapy with non-opioids and the need for opioid risk assessment when prescribing. A family centered approach was recognized as the goal of practice.

**Conclusion:**

Clinicians are less comfortable prescribing opioids to children for at-home use and find pain assessment and lack of clear guidelines to be barriers to pain care. Knowledge translation strategies for safer practice and optimal acute pain management could support responsible and judicious opioid use.

## Introduction

Pediatric pain management is one of the biggest challenges in the emergency department (ED) for healthcare professionals,^1,2^ with 50–80% of children having pain as one of their presenting symptoms to the ED.^3–6^ The World Health Organization describes children’s pain management as a public health concern of high importance.^7,8^ WHO guidelines affirm that ibuprofen and acetaminophen should be considered as the first-line oral analgesia for mild pain in children, with adjuvant opioids added for moderate to severe pain.^7^

Recently, the Canadian Institute of Health Information has declared opioid-related harms as “an issue of increasing public health importance” and advocated for decreased prescription of opioids.^9^ At the same time, in response to the Opioid Crisis, Children’s Healthcare Canada has suggested that “quality of life through effective pain management must not be lost in the addiction conversation,” reminding us that compassionate clinical care often includes the appropriate use of opioids.^10^ In the United States of America, the Food and Drug Administration states that it is “reassessing its approach to opioid products with the goal of reducing the opportunities for opioid misuse and abuse while ensuring that its actions are properly targeted, evidence-based, and serve the medical needs of patients while recognizing the critical role that healthcare providers play in addressing this public health priority.”^11^ This same sentiment of balancing risk with appropriate treatment is further echoed by the Stanford-Lancet Commission.^12^ While lifetime risk of opioid use disorder increases with opioid prescription, the true risk of developing an opioid use disorder after the use of *short-term* clinical opioids remains unclear.^13^ These factors contribute to complicated decision-making for clinicians treating children in the ED. We aimed to understand physicians’ decision-making considerations when managing children’s pain in the ED.

## Methods

### Study Design

This study followed a qualitative methodology employing grounded theory to make sense of pediatric emergency physicians’ decision-making when prescribing opioids for acute pain management.^14,15^ This included their (1) considerations when prescribing opioids to ensure safe practice, (2) management approach to hypothetical scenarios of varying acute pain presentations in children, and (3) perceived facilitators and barriers to prescribing opioids. We used semi-structured, audio-recorded telephone interviews. The interview transcripts were then analyzed using constant comparative methods. This study received ethical approval from the University of Alberta’s Health Research Ethics Board (Pro00070560).

### Sample Size

In line with the qualitative methodology, we recruited until thematic saturation was achieved. Two additional interviews were conducted after new themes ceased to emerge, to confirm thematic saturation. We used purposeful sampling to ensure diversity in the representation of physician demographics in our study population (e.g., gender, clinical experience, province of practice, age).^16^

### Study Setting and Population

We recruited currently practicing pediatric emergency physicians from pediatric tertiary care centers across Canada with a minimum of 1 year of clinical experience; access to both an email address and phone were pre-requisites for study participation. Our exclusion criteria included physicians not currently licensed to practice and lack of proficiency in spoken English. The research was conducted through the Department of Pediatrics (University of Alberta).

### Recruitment Methods/Procedures

Recruitment occurred from December 2019 to January 2021, via snowball sampling.^17^ Each co-investigator (SA, NP, MF) supported the interviewer (GS) to approach one pediatric emergency physician colleague via email. Informed consent was obtained if the approached physician agreed to participate (Supplement S1). At the end of each interview, the participant was asked to suggest one or two colleagues to approach, ideally with different perspectives than their own. The team reviewed the demographic characteristics of the interviewed physicians on an ongoing basis to ensure diversity as described above in keeping with a purposeful sampling.

### Data Collection

Interviews were conducted over the telephone by a qualitative methods-trained interviewer. The interview script and topic guide were developed based on findings in previous literature and finalized via research team consensus. Interviews lasted 20–40 min. The interview opened with the collection of demographic information (participant age, gender, working site, and affiliations). After that, the format shifted to open-ended questions. The interviewer also occasionally asked specific questions regarding the following subjects, if not brought up by the participant: experience prescribing opioids for acute pain, comfort/confidence levels surrounding opioid prescription, barriers and facilitators to opioid use, side effects, social considerations, opioid crisis, and family influence (Supplement S2). Field notes were also maintained by the interviewer. Interviews were electronically recorded. Audio files were submitted to a professional transcription service (https://transcriptheroes.ca) within 48 h of interview completion to facilitate iterative adjustments to future interview scripts.

### Data Analysis

All interviews were audio-recorded and sent securely to a professional academic transcription service provider (trasncriptheroes.ca). Transcript documents were then compared against the audio recordings to ensure that the transcripts were faithful to the original recordings. The transcripts were then entered into NVIVO 12.2.0 (QSR International).

Results were analyzed with the use of grounded theory utilizing constant comparison methods to theorize from the ground of our data how pediatric emergency physicians engage in decision-making when prescribing opioids for children’s acute pain management.^14,15^ In this way, we engaged in more than a qualitative description or other form of basic descriptive analysis of participants’ responses as we sought to understand of different considerations interact with one another to affect decision-making.

**Figure 1.**
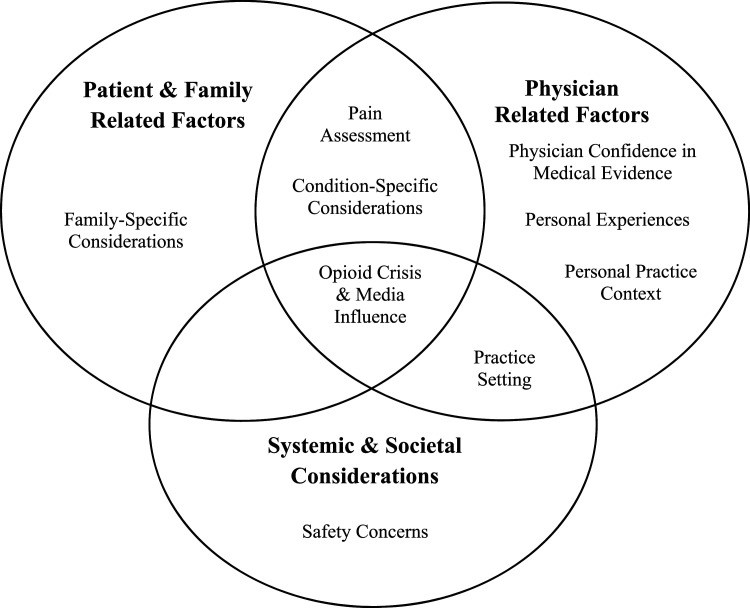
Conceptual model of “What influences Canadian pediatric emergency physicians when prescribing opioids for children with acute pain?” Note. The circles represent the interaction between the nine themes identified.

Coding was performed within the NVIVO environment concurrent to data collection involving open, axial, and selective coding. A single coder (GS) completed the initial open coding of the first few interviews to identify key phrases and concepts, and then organize these into subcategories and categories. The open codings of these interviews were compared for similarities and differences as an activity of constant comparison to ultimately inform creation of a preliminary coding scheme. This open coding performed by GS benefited from independent review and discussion of the transcripts by SA. As subsequent interviews were completed and transcribed, coding continued by GS with regular meetings with SA. Again, as an act of constant comparison, previous transcripts were re-coded when new themes emerged with the coding scheme revised to ensure the scheme encompassed the transcribed interviews. As the coding scheme became more mature, axial coding was completed to identify relationships and connections between codes. Finally, selective coding informed the refining of the existing coding to relate the codes together (i.e., as depicted in the graphic representation of the findings- Figure 1). Participants did not read the transcripts nor provide feedback on the analysis. All themes in the coding scheme were derived from the data (Supplement S3). The full study team also met to discuss the emerging themes; transcripts were re-coded based on their feedback as well.

## Results

A total of 11 included participants were included and proportionally represented the Canadian geographic regional population distribution: Eastern (10%), Central (45%), and Western Canada (45%). Participants were 73% (8/11) female and 27% (3/11) male. Their ages ranged from 30 to 55 years. Mean age was 43.2 years (SD ±6.03), in keeping with the current mean age of practicing pediatric emergency physicians in Canada.^18^

From analysis of the data, we identified how patient and family related factors, physician related factors, and systemic and societal considerations inform Canadian pediatric emergency physicians prescribing opioids for children with acute pain. Rather than being isolated factors, there are interactions and intersections between each (see Figure 1 for graphic depiction). We have organized our discussion section around these.

## Practice Setting and Outpatient Opioid Use

“I probably use them a lot more in the department when we’re using them intravenously or intranasally, and hardly at all at home when they’re being used orally.” ∼ Physician 3

Physicians consistently recognized the pivotal role opioids play in optimal analgesic care for acute pain presentations in the inpatient setting. However, while most physicians described comfort using opioids in the ED, they were not as confident when filling prescriptions for outpatient use at the time of discharge from the ED. Variability in approach depending on context was recognized by all physicians interviewed. While not everyone was reluctant to provide opioid prescriptions for at-home use, all physicians recognized differences when managing acute pain within the ED as opposed to at home, despite the similar pain pathophysiology in both settings. They also pointed out the lack of available treatment options when it came to prescribing opioids at discharge.

“When it comes to outpatient treatment with opioids there is a bit of an issue with being the most commonly used opioids with my understanding orally, is oral morphine, which has kind of a spotty record in terms of how efficacious it actually is, and it also has a significant side effect profile. So, I think to some extent the perception is also that we don’t have the same tools as effectively to treat outpatients’ pain with opioids.” ∼Physician 10

While some physicians mentioned they were not worried about opioid dependency as long as opioids were prescribed appropriately, most endorsed using very short duration outpatient opioid prescriptions, only rarely, and in very specific clinical scenarios.

## Condition-Specific Considerations

Participants identified medical considerations as a major element of decision-making. For example, the etiology of the acute pain such as musculoskeletal injury, acute appendicitis, or acute abdominal pain of undetermined etiology impacted decision-making. When the diagnosis was confirmed for a known highly painful condition (e.g., acute appendicitis), participants described no hesitation in providing opioids.

“ I mean if you have a suspicion – you know, this is kind of an ultra-severe pain like renal colic-type pain, biliary colic-type pain where the patient is in extreme distress, then yeah, I would definitely want to treat that.” ∼Physician 10

One participant highlighted how adequate analgesia could support diagnostic procedures and processes.

“Not the ones that have low pain, like ones that have mild to moderate pain. I don’t give them fentanyl before the ultrasound, but the one that has a bit of more severe pain, I think for the radiologist, their examination is made much more easy, and they get a better imaging of the appendix because the patient is able to relax or the patient is much more comfortable.” ∼Physician 1

Similarly, procedural analgesia was amongst the most agreed-upon indication for escalation of analgesic measures and utilizing opioids. This encompassed musculoskeletal injuries assessments/reductions and embedded foreign body removal. Furthermore, the availability of newer routes for opioid agents, namely—intranasal fentanyl, was widely recognized as a significantly impactful change for managing acute pain presentations.

“So, a lot of kids before, we were sending them to do the x-ray either with just Tylenol or Advil, but if you have a displaced fracture you can imagine, or elbow fracture, you have to extend the elbow to do the x-ray, it's quite painful. So, I feel that since we use that molecule [intranasal fentanyl], the kids come back from the x-rays much more quiet, more comfortable, they're not traumatized by the fact that they just had the x-ray. Same thing for appendicitis patients.” ∼Physician 1

Another frequent sub-theme discussed was the presence of other comorbidities that might make a clinician less likely to prescribe opioids, even when indicated. This included concurrent chronic disease, children with significant disabilities, and opioid-exposed patients.

“I mean certainly it would be, you know, the highly disabled kids, right, the ones who can get pathological fractures from osteopenia or, you know, they get a bowel obstruction or something like that. Those kids are really a non-communicator and probably get under-treated significantly.” ∼Physician 11

Another consideration was the expected duration of pain and its role in analgesic management. This impacted the decision to use opioids and was very evident in deciding which opioid to choose. Almost all participants agreed that opioids should be utilized as co-therapy, along with acetaminophen and ibuprofen, rather than monotherapy. Other options mentioned included nerve blocks, nitrous oxide, and procedural sedation, including the utilization of ketamine and the importance of employing physical and psychological interventions, such as ice packs, distraction via toys and digital devices, and caregiver comfort holds.

Another heavily emphasized sub-theme was considerations for special populations. For example, sickle cell disease and oncology patients were described as more likely to receive opioids compared to others.

“I think there are some very, very clear indications that essentially there shouldn’t be any reason to not prescribe opioids at discharge. That would be our oncology patients, sickle cell patients, especially patients who are already on narcotics.” ∼Physician 6

## Physician Confidence in Medical Evidence

Although ED physicians were aware of initiatives to enhance pain management, they simultaneously recognized deficiencies in confidence in the evidence and the need for further quality improvement, better knowledge translation, and more standardized practices for acute pain management.

“I think once there is evidence, then I think, you know…the next logical step is of course the quality improvement and knowledge translation piece of it. So, that’s where I think it’s probably important to standardize to some degree how people are practicing across pediatric EDs with respect to pain management, similar to other clinical areas that we have done that in.” ∼Physician 4

## Pain Assessment Challenges

Proper assessment of the degree of pain was highlighted as an essential yet challenging aspect of clinical decision-making when prescribing opioids in the ED. When gathering information on pain severity, validated child-reported pain severity scales were commonly employed tools in the ED.

“In the department I’m using - for kids who can do it, I’m using the scale out of ten as to when I consider opioids and as soon as I’m hearing seven out of ten or higher then I’ll offer [opioids].” ∼Physician 6

However, some providers stated their reliance on clinical impression to supersede such validated pain assessment tools.

“I have to admit, I should use pain scales more, but I don’t very often. I’ll often go with my clinical impression, or nature of the injury if it’s an evident injury.” ∼Physician 11

One of the biggest challenges mentioned was determining how best to assess pain severity in children. Many described challenges in choosing the best tool, as a lack of confidence in the evidence for pain measurement was raised. Overall, most participants reported relying on child-reported pain scores as a common standard of practice. The majority of our participants described switching to parental input, when the child was not able to give a direct response.

“I, as much as possible, try to just ask the question to the patient if they're able to answer. And if they're not, to the parent, as a surrogate, and just take that as an honest answer.” ∼Physician 9

## Patient and Family Perspectives

Family specific considerations included non-medical factors that might impact a physician’s decision when prescribing opioids. These encompassed both patient-dependent factors and caregiver-dependent factors.

“I think I’ve learned over the course of my career the importance of understanding from the patient and family perspective their preferences about pain, their thoughts about pain.” ∼Physician 7

A family-centered approach was recognized as the current gold-standard of practice unanimously, with points brought up such as parental concerns, culture, preferences, medical literacy, and hesitancy around opioids.

“If I think the child will really benefit from narcotic medication, I’ll explain to them why I think it’s safe and why they don’t need to be worried about it and why I think it would help them and give them that information. And if beyond that they still get to make the choice to be honest. I mean at that point if they’re still, “No we don’t want it”. I’m like, “OK you don’t want it, that’s a choice”. But I would sort of give them all the reasons why I think they do need it and why I think it’s not harmful or dangerous … there’s all kind of things that people worry about that are sort of realistic considerations.” ∼Physician 3

Others discussed included patient preferences, the child’s behavior in the ED, and the presence of either identifiable protective or high-risk factors. The latter included complex socio-economic situations, gender differences, and cultural background that might impact the interpretation of pain and any subsequent decision-making.

“Definitely, I think about it a little bit more looking at risk factors for dependency or, you know, kids who don’t have necessarily the support systems to support them taking, you know, narcotics and opiates, right. Like a lot of kids unfortunately have unstable social situations and, you know, just giving them a prescription of opiates to go home is going to be pretty unsafe possibly depending on, you know, a lot of factors.” ∼Physician 2

Patient age was identified as a significant variable affecting prescription of opioids, as well.

“… I would say still unfortunately younger children probably get undertreated for their pain since we are still not great at necessarily understanding their experience of pain versus anxiety.” ∼Physician 2

## Opioid Safety Concerns

“I think we need to be aware that it’s a powerful medication and it does potential have side effects, so we need to be cognizant of dose and potential for side effects.” ∼Physician 4

Physicians worried about higher-risk patients such as children or youth with known depression and suicidal ideation, lack of follow-up, misuse concerns, and existing substance use disorders.

“I think sometimes we will factor in previous life experiences or risk factors that - so for example I had a patient recently who one of my colleagues was hesitant to give opiates to because he had a past history of addiction to other drugs, depression, PTSD as well as a history of suicidal ideation. And so used those factors to think about is there another way to stop sending this kid home with pain medication that they may later get addicted to or have a risk for overdose.” ∼Physician 2

The risk of incorrect dosing or possible serious adverse events through accidental ingestions by other household occupants was also a concern for participants.

“If there’s a language barrier and parents aren’t understanding well, could they give a double dose? Could another kid in the house have access to the medication and take it and then, I don’t know, have a respiratory arrest because of my prescription?” ∼Physician 8

Finally, serious adverse events, as well as general medication safety profiles, concerned physicians when writing prescriptions for opioids.

## Personal Biases and Experiences

Participants noted how their own personal experiences might be impacting their clinical decision-making when it comes to opioid analgesics utilization. They acknowledged that these might include stereotypes and biases related to culture, race, and gender.

“I don’t know of paediatric-specific data for analgesia in people of various ethnicities. Certainly, Indigenous kids would be, to me, first and foremost in Canada. Black and other kids of colour as well. But I think Indigenous kids would probably be the ones more likely to experience, or most likely to experience bias in management of their pain.” ∼Physician 11

All physicians interviewed acknowledged their potential for harboring bias in assessing and treating pain, and this being an area for continued improvement.

“I guess my perspective has changed from the perspective of I believe the evidence that indicates that we’re not all that good at assessing other people’s pain and some of the sort of physical findings we sort of rely on such as ‘wow they’re not tachycardic so they can’t be in that much pain’ probably isn’t realistic.” … “but I think my biggest thought is that we either under-treat or delay the treatment, we wait until the patient is visibly uncomfortable or asks for analgesia quite often.” ∼Physician 3

## Personal Practice Context

All participants noted a considerable shift over the span of their career, either long or short, in how they treated pain mainly because of the educational efforts that have been made. Such changes were attributed to an increasing depth of physician knowledge and resources going along with the evolution of research and guidelines in the field.

“I think the sort of emerging evidence has probably been a bigger impact on how we practice because there are elements where I probably use narcotics more than I used to and there are areas where I use it less.” ∼Physician 3

Learning from and working alongside adult emergency-trained physicians, some of our participants felt that this helped them with their ultimate goal of better pain treatment while utilizing a harm-reduction approach. This included occasionally being involved in suboxone or methadone prescribing for detoxification programs.

Some systemic factors were also identified, such as obstacles to opioid prescriptions, including triplicate pad (controlled substance) prescription programs, differences in practices between primary and tertiary care centers, and the difference in safety measures between the outpatient (upon discharge from ED) versus the inpatient setting.

“There are of course operational barriers or system barriers, so what’s available on a particular setting, who can give it, the modality they have to give it to them, the scale of triage, where they’re able to weigh the child and give them IV; those kind of system and process level variables will also influence what patient; pediatric patients receive.” ∼Physician 7

## The Opioid Crisis and Media Influence

Geographical variability was noted in perspectives on the Opioid Crisis and its role in physician prescribing practices, with some deeming the crisis almost non-existent in certain parts of Canada and others indicating it was a daily consideration. All our participating emergency physicians agreed that there were increased family concerns and a need for parental and patient education on the safety of appropriate medical use of opioids for pain management. Most physicians described that they felt well-versed in opioid dependency risk. They indicated that the Opioid Crisis had minimally impacted their decision-making when considering opioids for acute pain management in the ED.

“I don’t think the Opioid Crisis has changed; that in the ED for the acute treatment, like when you see a patient that is suffering, whether you will give him morphine or not, I don't think it has changed.” ∼Physician 1

No perceived need to change practice due to the opioid crisis was mainly attributed to participants’ beliefs in already having adopted a harm-reduction approach or already having minimal opioid prescribing practices.

“I’ll say I don’t think it’s altered my practice, I never used opioids, I mean I never had the opportunity to use opioids even that much, besides for kids with pain in the ED; so, a pretty specific setting where I never used them a lot anyway. So, it hasn’t changed my practice.” ∼Physician 11

Interestingly, most participants reported increased family concerns specifically related to fentanyl use.

“I think we need to be aware of people’s perceptions of narcotics especially narcotics and just, you know, fentanyl’s got itself a bad rep in the press, rightly so when used as a recreational drug, but it’s certainly making some parents hesitant about it as a medical drug as well if you don’t know. You know it’s a very safe medical drug. So, I think we need to be aware of those elements.” ∼Physician 3

Finally, some physicians outlined some of the positive impacts social media has had on educating the general patient population regarding appropriate and safer opioids and analgesia.

## Discussion

**Figure 2.**
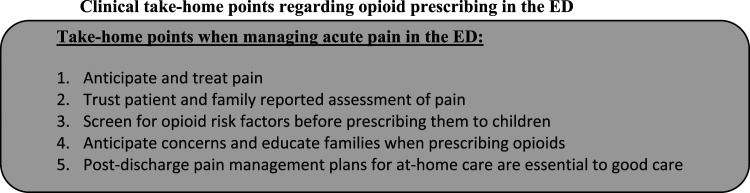
Clinical take-home points regarding opioid prescribing in the ED.

Pediatric emergency clinicians are key stakeholders in responsible opioid prescribing for children who are seen in pediatric hospitals with accidental injuries and acute illness. Our respondents highlighted the importance and benefits of anticipation and early pain management; this included anticipating any family concerns regarding opioids and trusting the patient and their family’s pain assessment. The importance of adequately managing pain during and after ED discharge was also highlighted, as were the feelings of greater comfort utilizing opioids in the ED setting rather than prescribing for at-home use. Finally, recognizing that social and demographic considerations, such as family risk factors for opioid use disorder, are not exclusive to adults and must be considered with children as well. Five key clinical implications are outlined below. (Figure 2):

### Anticipate and treat pain early

The importance of anticipation and initiation of early treatment of acute pain in the ED was highlighted as an essential aspect of pain management, irrespective of underlying etiology. Participants noted that while some had early pain management facilitated due to pre-existing protocols (i.e., sickle cell disease), others with not-yet-determined causes of pain would still benefit from timely pain management. This can be aided by implementing pre-authorized, nurse-initiated pain management protocols for acetaminophen and ibuprofen, and mandatory pain assessment protocols to allow for the use of opioid analgesia.^19–23^ A previous survey of Canadian pediatric emergency physicians about their willingness to prescribe opioids to children in the ED identified the top barriers to prescribing opioids in a timely manner as parental reluctance, lack of clear guidelines for pediatric opioid use, and concern about adverse effects; all of which were similarly represented in our participant interviews.^24^ Furthermore, similar to our study findings, ED physicians appeared minimally concerned about physical dependence, addiction risk, and the current Opioid Crisis when prescribing opioids to children. They felt they were generally already using opioids judiciously.^24^

### Trust Patient and Family Reported Assessment of Pain

Accurate pain assessment in children can be one of the most challenging tasks for a physician. This resulted in some difficulty in choosing the best approach when assessing a child’s degree of pain in the pediatric ED. Previous literature has established that the best means to properly assess a child’s degree of pain is through self-report.^25^ It is imperative to attend to a child in order to establish a “child-centered focus” in managing pediatric acute pain with an age-appropriate validated pain assessment tool.^26,27^ While some of our participants felt that healthcare professionals were better situated to assess the child’s pain over the child or child’s caregiver/parent, this misconception directly contradicts current understanding, which suggests that a child’s caregiver/parent provides a more accurate assessment of pain and management than a healthcare professional.^28^

### Screen for Opioid Risk Factors Before Prescribing Them to Children

Participants recognized the utmost importance of risk assessment when prescribing opioids for children. Participants noted that not all pediatric patients are opioid-naïve and many live with risk factors for opioid use disorder. A recent systematic review reports a likely correlation between therapeutic opioid use and future non-medical opioid use, although a direct link to short-term opioid use could not be determined.^13^ As such, ED physicians should continue to thoroughly consider non-opioid analgesic options and educate patients and caregivers around safe, prudent, and appropriate use of opioids and possible signs of misuse when prescribed.^13^

### Anticipate Concerns and Educate Families When Prescribing Opioids

Ongoing and open communication with patients and families was identified as vital to optimal outcomes when safely managing acute pain in the ED. Taking a proactive approach in acknowledging family concerns and hesitations regarding opioid utilization in pain treatment ensures a more transparent and less stressful experience for everyone involved. This includes discussing the need for opioid use when clinically indicated and the safety measures employed when using opioids in a regulated healthcare setting. This extends to appropriate discharge education and instructions when prescribing opioids as an outpatient with a clear and easy-to-comprehend follow-up plan. Previous studies commented on how some patients stated that they would have benefited from additional advice in the ED.^29^ Openly discussing the follow-up plan and prognostic expectations may help ease some of these concerns; written instructions or online resources at discharge can also be beneficial.^30^ Education should include advice on how to combine opioids with non-opioid pain medications, the use of opioids for a short time only, monitoring for side effects, safe storage of the opioids, never sharing opioids with anyone else, and how to dispose of opioids safely.^31^

### Post-Discharge Pain Management Plans for at-Home Care are Essential to Good Care

There is a continued need for adequate pain treatment in the at-home setting. Previous studies have described how children with fractures experience significant pain at home, especially in the first 3 days following care in the ED.^1, 32–34^ Participants voiced the importance of a well-thought outpatient pain management plan, but also described a relative discomfort in prescribing opioids for at-home use, when compared to the in-hospital setting. Given the impacts of inadequately managed pain as an outpatient can negatively impact a child’s functioning through altered sleep, appetite, play/sport/school participation, and mood,^35^ further focus on training and knowledge-sharing with clinicians to support their prescribing for at-home use is required.

## Limitations

Limitations included generalizability due to the sample size, potential selection and respondent bias, and the inclusion of only English-speaking physicians. Although we reached thematic saturation, it is important to acknowledge that our recruitment strategy was in part based on convenience, with participants all practicing in large Canadian hospitals staffed by providers trained or experienced in pediatrics. Participants represented one-third of Canada’s pediatric emergency departments, and their geographical distribution reflected that of pediatric emergency practitioners across Canada; however, the findings cannot necessarily be generalized to non-pediatric (urban and rural) emergency settings, where the majority of Canadian children are first treated for injury and illness. Still, serious illness and injury is almost always referred to one of Canada’s 15 pediatric hospitals, where their tertiary care would generally begin in the emergency department, as the entry point to the hospital. While the snowball method of recruitment can introduce some bias towards similarity in perspectives, our participants were explicitly asked to identify both colleagues with similar and differing views or practice patterns from themselves, in order to minimize this. Lastly, all responses were self-report, and actual practice patterns were not verified, However, given that our goals were to understand perspectives rather than measure behaviors, this should have minimally impacted our results.

## Conclusions

Both patient-specific and personal social factors and experience are involved when clinicians prescribe opioids to children. Further, taking a proactive approach in acknowledging family concerns and hesitations regarding opioid utilization in pain treatment ensures a more transparent and less stressful experience for everyone involved. Lastly, there is a need for quality improvement when considering outpatient pain management. This can help inform knowledge translation strategies for safer practice and optimize acute pain management in pediatrics. When considering opioids for acute pain management in children, key considerations include the importance of: (1) Anticipating and treating pain early; (2) Trusting patient and family reported assessment of pain; (3) Screening for opioid risk factors before prescribing them to children; (4) Anticipating concerns and educating families when prescribing opioids’; and (5) Post-discharge pain management plans for at-home care.

## Supplemental Material

Supplemental Material - What influences physician opioid prescribing for children with acute pain? Click here for additional data file. Supplemental Material for What influences physician opioid prescribing for children with acute pain? by George Slim, Michael van Manen, Megan Fowler, Naveen Poonai, and Samina Ali in British Journal of Pain
