# Supplementary material for: What influences physician opioid prescribing for children with acute pain?
Source: Br J Pain. 2022 Dec 15;17(2):195–205. doi: 10.1177/20494637221146421 (PMC10088422; doi:10.1177/20494637221146421)
Supplement: Supplemental Material - What influences physician opioid prescribing for children with acute pain? [file sj-pdf-1-bjp-10.1177_20494637221146421.pdf]

Data Supplement S1: Informed Consent Form:

Canadian Pediatric Emergency Physicians' Perspectives on Opioid Prescribing for Children with Acute Pain

[redacted]

Purpose and Procedure:

As a member of Pediatric Emergency Research Canada (PERC), you are being invited to participate in a PERC-endorsed telephone interview. The purpose of this interview is to try to better understand Pediatric Emergency Medicine Physicians' opioid prescribing practices for children with acute pain in the emergency department (ED), and at discharge.

You will be asked questions regarding your demographics and your thoughts on opioids prescriptions for acute pain in pediatric emergency departments. This may or may not be followed up by some probing questions that might involve knowledge of potential risks and benefits of short-term opioid use in children, pain management practices for hypothetical scenarios and perceived barriers and facilitators to prescribing opioids. The results of this study will be used to help guide development of educational forums, research and protocols for pain management in pediatric ED.

Voluntary Participation:

This interview has been piloted and should take 30-45 minutes of your time. Your participation is voluntary, and you are free to decline to answer any questions on the interview. If, at any time, you decide you no longer wish to participate, simply let the interviewer know and they shall

wrap it up swiftly. Identifying data will be stored separately from the interview transcripts. This study has been approved by the Research Ethics Board (REB) of the [redacted]

Benefits & Risks:

There are no anticipated risks or benefits to you related to participating in this study.

Confidentiality:

No data relating to this study that includes your name will be published by the researchers. The telephone interview will be recorded however this will be securely stored in our password protected computers. These recordings will be transferred to another company securely in order to be transcribed. All individuals handling the files have signed confidentiality agreements.

- Your information will not be shared with anyone outside of the research team.
- All data will be kept for a minimum of five years in accordance with University of Alberta policy.
- Your name will not be attached to your information.
- Your name will never be used in any presentations or publications of the study results.

Consent will be implied by taking part of the interview. If you have any questions or concerns regarding your rights as a participant, or how this study is being conducted, you may contact the [redacted] Research Ethics Office at [redacted]. This office has no affiliation with the study investigators. If you have any other questions regarding this study, please contact:

[redacted]

Data Supplement S2: Interview Script:

Hello! My name is ____ and I am a researcher from the [redacted]. Thank you for taking the time today for this interview and for being part of our study. We want to learn about how the thoughts and circumstances that affect your day-to-day decision-making when encountering children presenting with acute pain in the emergency department. There are no right or wrong answers. I would like to hear about what you really think and how you really feel. If it’s okay with you, I will be electronically recording our conversation since it’s hard for me to write everything down while we talk. The reason I’m recording is so that I can go back and re-listen to our conversation to learn from what you say. Everything that you say will be kept confidential, meaning that only myself and my research teammates will be aware of what you say. If at any time you feel that you don’t want to keep talking with me, we can stop the interview.

Do you have any questions before we begin?

Gather basic information:

For the records, can you please state your full name?

And just so we have our information correct; you are a practicing pediatric emergency physician at (institution’s name)?

Introductory questions:

What are your thoughts on opioids prescriptions for acute pain in pediatric emergency departments?

What are your usual thoughts and considerations when considering opioids for acute pain management?

Probing Questions:

What unique factors should emergency room physicians consider when prescribing opioid compared to other medications?

What ought a good emergency physician consider when prescribing opioids to ensure safe practice?

Could you describe a time when you felt uncomfortable prescribing opioids? What lead you to feel uncomfortable? What was the outcome?

Consider the following situation: Parents bring in an 8 years-old with acute abdomen pain. The clinical exam, bloodwork, and x-rays are reassuring yet you decide to observe for a few hours before discharge. How would you manage the pain? What would factor into your decision-making to prescribe opioids?

What are your personal experiences with severe pain?

Do you have specific preference when deciding on analgesics for children?

What are your thoughts on the Opioid Crisis?

Topic guide (if brought up by other physicians in previous interviews/surveys):

What are your responsibilities to families when prescribing opioids?

How would you describe your confidence level regarding opioids for acute pain management in children?

What are your dosing preferences, if any, when prescribing opioids to children?

Do you think that age is a factor affecting your decision-making?

Is safety a big concern when prescribing opioids for acute pain management? What constitutes safety?

Do social considerations happen to be one of the factors you consider when prescribing opioids?

Data Supplement S3: The Codebook Tree:

1.0 Treatment Setting
1.1 Inpatient Analgesia
1.1.1 Currently implemented protocols
1.1.2 Prescription frequency
1.1.3 Protective factors for inpatient analgesia
1.1.4 Comfort with available tools
1.1.4.1 Continuous observation
1.1.4.2 More comfort because of safety
1.1.4.3 Nursing availability
1.1.5 Time-sensitivity
1.1.5.1 Pre-set orders
1.2 Outpatient Analgesia
1.2.1 Discharge analgesia prescriptions
1.2.2 No opioids on discharge
1.2.2.1 Barriers
1.2.2.2 opioid alternatives - NSAIDS, Tylenol
1.2.3 Rarely opioids on discharge
1.2.3.1 Short Duration
1.2.3.2 Situational
1.2.4 Yes, for opioids on discharge
1.2.4.1 Discharge Education
1.2.4.1.1 Handouts
1.2.4.1.2 Return to care instructions
1.2.5 High risk medications
1.2.5.1 Are all Opioids High Risk
**2.0 Family Specific Considerations**
2.1.1 Parents Dependant Factors
2.1.1.1 Family-centered approach

2.1.1.2 Parental Concerns
2.1.1.2.1 Fentanyl Specific Concerns
2.1.1.2.2 Opioid crisis
2.1.1.2.3 Geographical variability
2.1.1.3 Parental Education
2.1.1.3.1 Parental Health Literacy
2.1.1.3.2 prior to administration
2.1.1.3.3 safety in hospital compared to street use
2.1.1.4 Parental Preferences
2.1.1.4.1 Parental Adherence
2.1.1.4.2 Parental comfort
2.1.1.4.3 Parental fear of masking underlying etiology
2.1.1.4.4 Parental hesitancy
2.1.1.4.5 Parental interpretation of pain severity
2.1.1.4.6 Parental Negative prior experiences
2.1.1.4.7 Reluctance even prior to Opioid Crisis
2.1.1.4.8 Substitute decision maker
2.1.2 Patient Dependant Factors
2.1.2.1 Demographic variables
2.1.2.1.1 Cultural background
2.1.2.1.2 Gender
2.1.2.1.3 Patient age group
2.1.2.1.4 ability to assess pain severity
2.1.2.1.5 age based risk for opioid dependency-diversion
2.1.2.1.5.1 teenage years higher risk
2.1.2.1.6 age impact on pain perception
2.1.2.1.7 age-based preferences for analgesia
2.1.2.1.7.1 hesitancy prescribing opioids to infants under 1 year of age
2.1.2.1.8 available routes per age group
2.1.2.1.9 Development

2.1.2.1.10 Follow-up potential
2.1.2.1.11 Medication safety risk
2.1.2.1.11.1 higher risk in younger populations
2.1.2.1.12 need for dosage adjustment for special age groups - infants
2.1.2.1.13 No noticeable impact
2.1.2.1.14 Speech development
2.1.2.1.15 Racial background
2.1.2.1.16 Socio-economic status
2.1.2.2 Negative Patient Factors
2.1.2.2.1 Current Drug Addiction
2.1.2.2.2 Lack of family support
2.1.2.3 Patient behaviours
2.1.2.3.1 Anxiety
2.1.2.4 Patient Preferences
2.1.2.4.1 Negative prior experience
2.1.2.4.2 PTSD
2.1.2.5 Positive Patient Factors
2.1.2.5.1 Strong family support
2.1.3 Social Status Concerns
2.1.3.1 Family History of Substance Use
2.1.3.2 Not concerned
2.1.3.2.1 adequate analgesia more important
2.1.3.2.2 inpatient
2.1.3.2.3 outpatient
2.1.3.3 Yes, concerned
2.1.3.3.1 changes in dose, frequency, and agent used
2.1.3.3.2 inpatient
2.1.3.3.3 outpatient
2.1.3.3.4 diversion risk
**3.0 Medical Considerations**

3.1 Analgesia Options
3.1.1 Analgesia Routes
3.1.2 Intranasal
3.1.3 Intravenous
3.1.4 Local
3.1.5 Oral
3.1.5.1 Liquid
3.1.5.2 Tablet
3.1.6 Transdermal
3.1.7 Analgesic Medications and Interventions
3.1.8 Acetaminophen
3.1.9 Fentanyl
3.1.9.1 Pros
3.1.10 Hydromorphone
3.1.11 Intravenous sedation
3.1.11.1 General Anaesthesia
3.1.11.2 Ketamine
3.1.12 Morphine
3.1.13 Nerve Blocks
3.1.14 Nitrous oxide
3.1.15 Non-pharmacologic interventions
3.1.16 NSAIDS
3.1.17 Opioids
3.1.17.1 Adjunct
3.1.17.2 First Line
3.1.18 Oxycodone
3.1.19 Sucrose
3.2 Common Acute Pain Presentations
3.2.1 Acute Abdominal Pain
3.2.2 Functional vs Organic

3.2.3 Appendicitis
3.2.4 better imaging if pain well controlled
3.2.5 Musculoskeletal injuries
3.2.6 Osteogenesis Imperfecta
3.2.7 Pre-imaging
3.3 Comorbidities
3.3.1 chronic lung disease
3.3.2 highly disabled children - difficulty communicating
3.3.3 Known history of substance use-dependency
3.3.4 amphetamine
3.3.5 need for higher dosage to control pain
3.3.6 Non-opioid naive patients
3.3.7 Osteopenia
3.4 Expected duration of pain
3.4.1 Fentanyl - shorter and faster pain relief
3.4.2 Morphine - longer duration of relief
3.5 Procedural analgesia
3.5.1 Foreign body disimpaction
3.5.2 IV line insertion
3.5.3 Lumbar puncture
3.6 Special populations
3.6.1 Burns
3.6.2 chronic use of analgesia
3.6.3 Tylenol
3.6.4 Comorbidities and Medication Interaction Considerations
3.6.5 Dental Pain
3.6.6 Herpetic gingivostomatitis
3.6.7 NAS
3.6.8 Oncology patients
3.6.9 Post-tonsillectomy

3.6.10 ENT preference against traditional NSAIDS
3.6.11 outpatient morphine
3.6.12 Sickle Cell Disease
**4.0 Pain Assessment**
4.1 Child reported
4.2 Clinicians' interpretation of pain severity
4.3 Cultural variations
4.4 Multifactorial input
4.5 Pain scale - numerical
4.6 Poor assessment of pain severity in children
**5.0 Physician Confidence in the Evidence**
5.1 Concerns of Being Outlier in Practice
5.2 More research needed
5.2.1 Knowledge Translation
5.2.2 clear guidelines on opioids for outpatient analgesia
5.2.3 Need for further data on racial biases and discrimination
5.2.4 understanding what high-risk medications are
5.3 Quality improvement
5.3.1 New Protocols
5.3.2 creating new protocols for speciality populations - e.g. sickle cell disease
5.3.3 Creating new protocols including safe opiates (intranasal fentanyl)
5.3.4 ongoing training for emerging challenges
5.3.5 support for children known for substance use
5.3.5.1 Safe injection sites in hospitals
5.3.6 Standardize opioid prescriptions in pediatrics
5.4 Suggestions For Future
5.4.1 Emerging challenges
5.4.2 Treating withdrawal in known substance use
5.4.2.1 Amending the Mental Health Act to encompass drug withdrawal safety
5.4.2.2 Knowledge deficit in management of withdrawal in pediatrics

5.4.2.3 not a common presentation in pediatrics
**6.0 Physician Personal Experiences**
6.1 Physician biases
6.1.1 Cultural biases
6.1.2 Gender biases
6.1.3 Physicians' comfort
6.1.4 Racial biases
6.1.4.1 children of colour (black)
6.1.4.2 indigenous
6.1.5 Stereotypical Biases
**7.0 Physician Professional Context**
7.1 Physician knowledge and resources
7.1.1 appropriate dosing
7.1.2 E-Formularies
7.1.3 Importance of accurate assessment of pain
7.1.4 Pain as the 5th vital sign
7.1.5 Multidisciplinary Input
7.1.6 Anaesthesia
7.1.7 Expert recommendations
7.1.7.1 Adolescent Medicine Specialists
7.1.7.2 Adult-trained Emergency Medicine Physicians
7.1.7.3 ENT specialists
7.1.8 Nursing
7.1.8.1 nurses change in perspective
7.1.8.1.1 more timely pain control
7.1.9 Pain team
7.1.10 Research Assistants
7.1.11 Physician background training
7.1.12 Recency of Training
7.1.13 Research and Guidelines

7.1.14 Deficiency in evidence-based guidance
7.1.15 Emerging evidence provide more comfort in practice
7.1.16 Evidence on difficulty of accurately assessing pain severity in children
7.1.17 Literature supporting opioid alternatives
7.1.18 Local committees
7.1.19 Nurse initiated protocols
7.1.20 Recent Research
7.1.21 Trauma-informed care
7.2 Physician perspective changes
7.2.1 acute pain vs chronic pain management
7.2.2 Addressing underlying etiology
7.2.3 Aim for better use of alternative analgesia options
7.2.4 Nerve Blocks
7.2.5 Better pain control is the goal
7.2.6 Better understanding of analgesic medications
7.2.7 Better understanding of pain
7.2.8 Changes in Standard of Practice
7.2.9 Changes in place to opioid prescribing practices
7.2.9.1 availability of new routes
7.2.9.2 avoiding alternative painful routes - intravenous
7.2.9.3 better safety protocols
7.2.9.4 Less duration
7.2.10 Current standard of practice
7.2.10.1 Switch away from Codeine
7.2.11 decision-making precedes full data collection
7.2.12 Geographical variability
7.2.13 No fear of masking underlying pain, diagnosis
7.2.14 Harm-reduction approach
7.2.15 Higher emphasis on clinical guidelines recommendations
7.2.16 Individualized approach

7.2.17 knowledge of underlying disease process
7.2.18 affects decision making - no (pain should be treated adequately regardless of underlying etiology)
7.2.19 affects decision making - yes
7.2.19.1 If unclear, likely not needing opioids unless significant underlying etiology
7.2.19.2 may consider further investigations prior
7.2.20 Less Opioids
7.2.21 More holistic approach
7.2.22 More Opioids
7.2.23 No change
7.2.24 physician preferences
7.2.25 Physicians’ current stance on prescribing opioids
7.3 Physicians Dependant Factors
7.4 Systemic Factors
7.4.1 Obstacles to Opioid Prescriptions
7.4.2 Knowledge translation gaps
7.4.3 Limited options as outpatient vs inpatient
7.4.4 More side effects associated with oral options than other routes
7.4.5 System barriers
7.4.5.1 Available resources
7.4.5.2 Available medical personnel
7.4.5.3 Clinical skillset
7.4.5.4 Scale of triage
7.4.6 Triplicate Pad availability
7.4.7 Triplicate prescription pads
7.4.8 Primary vs Tertiary centers
7.4.9 Added importance of clinical guidelines
7.4.10 Resources available
7.4.11 Standards of practice
7.4.12 Safety Measures
7.4.13 Adverse events reporting system

7.4.14 appropriate indication and dosage
7.4.15 clear administration instructions
7.4.16 College mandated CME - prescribing opioids, benzos, stimulants
7.4.17 Education on potential side effects
7.4.18 Familiarity with 1-2 preferred choices of narcotic analgesia
7.4.18.1 downside to physician comfort with preferred choices
7.4.19 Follow-up appointment
7.4.20 High-risk assessment tools
7.4.20.1 no - not used
7.4.20.1.1 knowledge deficit
7.4.20.2 yes - used
7.4.21 Keep hospitalized longer
7.4.22 New protocols
7.4.23 Nursing team checks
7.4.24 Pharmacist role
7.4.25 Reassessment
7.4.26 Return to care instructions
7.4.27 Short duration prescriptions
7.4.28 triplicate pad prescriptions
**8.0 Safety Concerns**
8.1 High Risk Patient Profile
8.1.1 Depression and Suicidal Ideation
8.1.2 Lack of follow-up
8.1.3 Recreational Concerns and Existing Addiction
8.1.4 TSD
8.2 Incorrect Dosing
8.2.1 Language Barrier
8.2.2 Pharmacist Errors
8.2.3 Prescription Errors
8.3 Side Effects

8.3.1 Accidental Ingestions by Other Household Occupants
8.3.2 Lethal Side Effects
8.3.3 Respiratory arrest
8.3.4 Medication Safety
8.3.5 NSAIDS induced GI bleeds
**9.0 The Opioid Crisis and Media Influence**
9.1 Geographical variability
9.2 Opioid Crisis and Parental Concerns
9.2.1 Fentanyl Specific Concerns
9.2.2 Negative prior experiences
9.2.3 Opioid crisis
9.2.4 Parental Education
9.2.5 prior to administration
9.2.6 safety in hospital compared to street use
9.3 Opioid Crisis and Physician Concerns
9.3.1 impact on physician practices
9.3.2 concerns about reports to the college
9.3.3 less outpatient opioid analgesia prescriptions
9.3.4 Opioid Crisis and Dependency Risk
9.3.5 Age variability
9.3.5.1 Adults problem
9.3.6 geographical variability
9.3.7 No - Not concerned
9.3.8 Opioid Crisis
9.3.9 Yes - concerned
9.4 Standard and Social Media
9.4.1 impact on physicians
9.4.2 change in education
9.4.2.1 more patient and parent education in advance
9.4.3 change in practice

9.4.3.1 inpatient
9.4.3.1.1 less
9.4.3.1.2 more
9.4.3.1.3 no change
9.4.3.2 less
9.4.3.3 more
9.4.3.4 no change
9.4.3.5 outpatient
9.4.3.5.1 less
9.4.3.5.2 more
9.4.3.5.3 no change
9.4.4 utilizing social media in physician CME
9.4.5 minimal impact
9.4.6 negative impact
9.4.7 positive impact
